# PW01-039 – Long-term efficacy of anakinra in SoJIA patients

**DOI:** 10.1186/1546-0096-11-S1-A92

**Published:** 2013-11-08

**Authors:** A Naselli, J Tibaldi, A Accogli, A Buoncompagni, S Viola, S Signa, P Picco, A Ravelli, A Martini, M Gattorno

**Affiliations:** 1 Pediatria 2, IRCCS, Istituto Giannina Gaslini, Genoa, Italy

## Introduction

Recombinant IL-1 receptor antagonist (anakinra) is an effective treatment in a subgroup of systemic onset JIA (SoJIA). So far no information is available on the long term follow-up of SoJIA patients treated with anakinra.

## Objectives

To analyse the long term follow-up of SoJIA patients treated with Anakinra in a single tertiary referral center.

## Methods

Since 2005, 34 SoJIA patients (19 M, 15 F) were treated with anakinra at the staring dose of 1-2 mg/kg/die. Complete response was defined as the absence of systemic and articular manifestations and normal acute phase reactants at follow-up, with anakinra as a monotherapy. Other patients were considered as partial responders (still in anakinra with evidence of disease activity) or non-responders (withdrawn of Anakinra due to inefficacy or severe side effects).

## Results

At baseline, the mean age was 8.4 year (range 1-17 years) with mean disease duration of 3.05 years (3 months-10.2 years). All patients had active arthritis (mean number of active joints 12.3, range 1-80), 28/34 had fever, 20/34 had skin rash. Failure of anti-TNF treatment or DMARD was observed in 11/34 and 23/34 patients respectively. Ongoing steroid treatment 33/34 patients (mean prednisone mg 0.87/kg/day, range 0.1- 3). The mean follow-up was 4.02 year (range 1.05 - 6.16). At the last follow-up 13 patients (38%) were complete responders, 5 (14%) partial responders and 16 (48%) non responder. Among complete responders, 4 patients withdrawn anakinra without relapses after a mean of 3 years of treatment, 7 are in remission using anakinra as mono-therapy, 2 patients were switched to anti IL-1 monoclonal antibody with a full response. Despite the good control of their disease 11/13 displayed at least one relapse of their disease during the follow-up with a total of 22 relapses (range 1-4 for patient). In 16 non responders patients subsequent treatments were canakinumab (1 patient), tocilizumab (5 patients), or combined immunosuppressive treatment and/or anti-TNF (10 patients), with variable response. Adverse events and complications: 13 Anakinra-treated patients had skin reactions of variable intensity and duration, in 7 patients hitching was also present without an evident skin rash. Six patients (3 responder and 3 non responders) developed a MAS during follow-up. Two non-responders patients died for acute bacterial meningitis and multi-organ failure after an episode of MAS. As previously observed, responders patients confirmed to have an higher number of active and limited joints at baseline (p = 0.006) and higher WBC and neutrhophils count (p = 0.002). These results were confirmed in the newly enrolled patients. In this latter group responder patients displayed a significantly shorter disease duration in respect to non responder patients (p = 0.03).

## Conclusion

In responder SoJIA patients Anakinra confirm to be an effective and safe drug in the long term. The use of Anakinra is still able to dissect two distinct populations of SoJIA patients on the basis of the presence or not of a severe joint involvement with a chronic polyarticular course.

## Disclosure of interest

None declared

